# Full-length transcriptome analysis of maize root tips reveals the molecular mechanism of cold stress during the seedling stage

**DOI:** 10.1186/s12870-022-03787-3

**Published:** 2022-08-13

**Authors:** Li Xuhui, Chen Weiwei, Lu Siqi, Fang Junteng, Zhu Hang, Zhang Xiangbo, Qi Yongwen

**Affiliations:** 1 Institute of Nanfan & Seed Industry, Guangdong Academy of Science, Guangzhou, 510316 Guangdong China; 2 Guangdong Laboratory for Lingnan Modern Agriculture, Guangzhou, 510642 Guangdong China; 3 College of Agriculture and Biology, Zhongkai University of Agriculture and Engineering, Guangzhou, 510325 Guangdong China; 4 College of Agriculture, Yangtze University, Jingzhou, 434025 Hubei China

**Keywords:** Cold stress, Full-length transcriptome, Seedling stage, Maize, ROS, Amine

## Abstract

**Background:**

As maize originated in tropical or subtropical zones, most maize germplasm is extremely sensitive to low temperatures during the seedling stage. Clarifying the molecular mechanism of cold acclimation would facilitate the breeding of cold tolerant maize varieties, which is one of the major sustainability factors for crop production. To meet this goal, we investigated two maize inbred lines with contrasting levels of cold tolerance at the seedling stage (IL85, a cold tolerant line; B73, a cold sensitive line), and performed full-length transcriptome sequencing on the root tips of seedlings before and after 24 h of cold treatment.

**Results:**

We identified 152,263 transcripts, including 20,993 novel transcripts, and determined per-transcript expression levels. A total of 1,475 transcripts were specifically up-regulated in the cold tolerant line IL85 under cold stress. GO enrichment analysis revealed that 25 transcripts were involved in reactive oxygen species (ROS) metabolic processes and 15 transcripts were related to the response to heat. Eight genes showed specific differential alternative splicing (DAS) in IL85 under cold stress, and were mainly involved in amine metabolism. A total of 1,111 lncRNAs were further identified, 62 of which were up-regulated in IL85 or B73 under cold stress, and their corresponding target genes were enriched in protein phosphorylation.

**Conclusions:**

These results provide new insights into the molecular mechanism of cold acclimation during the seedling stage in maize, and will facilitate the development of cultivars with improved cold stress tolerance.

**Supplementary Information:**

The online version contains supplementary material available at 10.1186/s12870-022-03787-3.

## Background

Maize (*Zea mays* L.) is one of the most important cereal crops. However, most maize germplasm is sensitive to low temperature, as it originated in tropical or subtropical zones [1, 2]. At high latitudes and altitudes, there is a high risk of exposure to cold stress for maize, which reduces plant growth, and grain yield and can even cause death [3, 4]. Especially at the seedling stage, the root, as the fundamental organ for water and nutrient absorption, would affect the growth and development of maize seedlings at suboptimal temperatures [5]. Therefore, understanding the root response to cold stress at the molecular level is of great importance for developing cold-resistant crops, which is one of the major sustainability factors for crop production.

Assessing gene expression patterns based on transcriptome sequencing data is an important way to elucidate the molecular mechanism of cold acclimation in plants. Many studies have shown the effectiveness of transcriptome analysis in understanding how altered expression contributes to complex traits, such as cold tolerance [6, 7]. Using transcriptome analysis, more than 100 regulons of CBF transcription factors were found to be rapidly induced under cold stress [8]. In addition to genes related to the CBF cold response pathway, genes associated with signal transduction, membrane part, response stimulus, plant hormone signal transduction, abiotic stress response and zeatin biosynthesis, were significantly differentially expressed in plants under cold stress according to a comparison of transcriptomes between genotypes with contrasting levels of cold tolerance [9–11]. In maize, a number of cold response genes have been identified, and the functions of these genes were found to be largely similar to those of other plants [12–20]. Although thousands of cold-responsive genes have been identified in plants, most of these studies mainly used the Illumina sequencing platform, which generates short reads (< = 150 bp), making it difficult to quantify expression at the transcript level.

Full-length RNA sequencing has recently become commercially available recently, and can be performed by Oxford Nanopore Technologies (ONT), Pacific Biosciences (PacBio) and others [21, 22]. These technologies enable single-molecule sequencing of complete individual RNA molecules or cDNA, and enabled the generation of full-length cDNA/RNA reads for transcripts up to 15 kb [23]. Thus, this technique permits efficient transcript assembly, the quantification of transcript levels and alternative splicing (AS) events, and a novel transcript identification [24, 25]. Using the ONT sequencing platform, Yu et al. (2021) identified 1390 transcripts in *P. damicornis* in response to different growing environments [26]. Utilizing the PacBio sequencing platform, a number of novel transcripts and genes were identified and 56 differentially expressed genes were found under drought/heat stress in rice [27]. Most maize genes contain multiple exons and introns, which can produce several transcripts [28]. Identifying the changes in transcript abundance under cold stress is crucially important for researchers. To date, few studies have focused on the full-length transcriptome in response to cold stress in maize. Therefore, to advance the knowledge of molecular mechanisms under cold stress, it is necessary to assess global full-length transcriptome changes under cold stress during the seedling stage in maize.

In this study, we screened two maize inbred lines with contrasting levels of cold tolerance at the seedling stage. To characterize different transcriptional responses in the root tips of the two lines under cold stress, the ONT sequencing platform was used on the 2 cm samples of root tips from seedlings before and after 24 h of cold treatment (10 °C). Due to the long-read length of ONT Nanopore-based RNA sequencing, the accuracy of transcript assembly was greatly improved, and per-transcript expression abundance was quantified and used for further differentially expressed transcript (DET) and alternative splicing (AS) event analysis. The results of this study provide important information regarding the molecular basis of cold acclimation and the future of cold tolerance breeding in maize.

## Results

### Effects of cold stress on the electrolyte leakage of roots

**Fig. 1:**
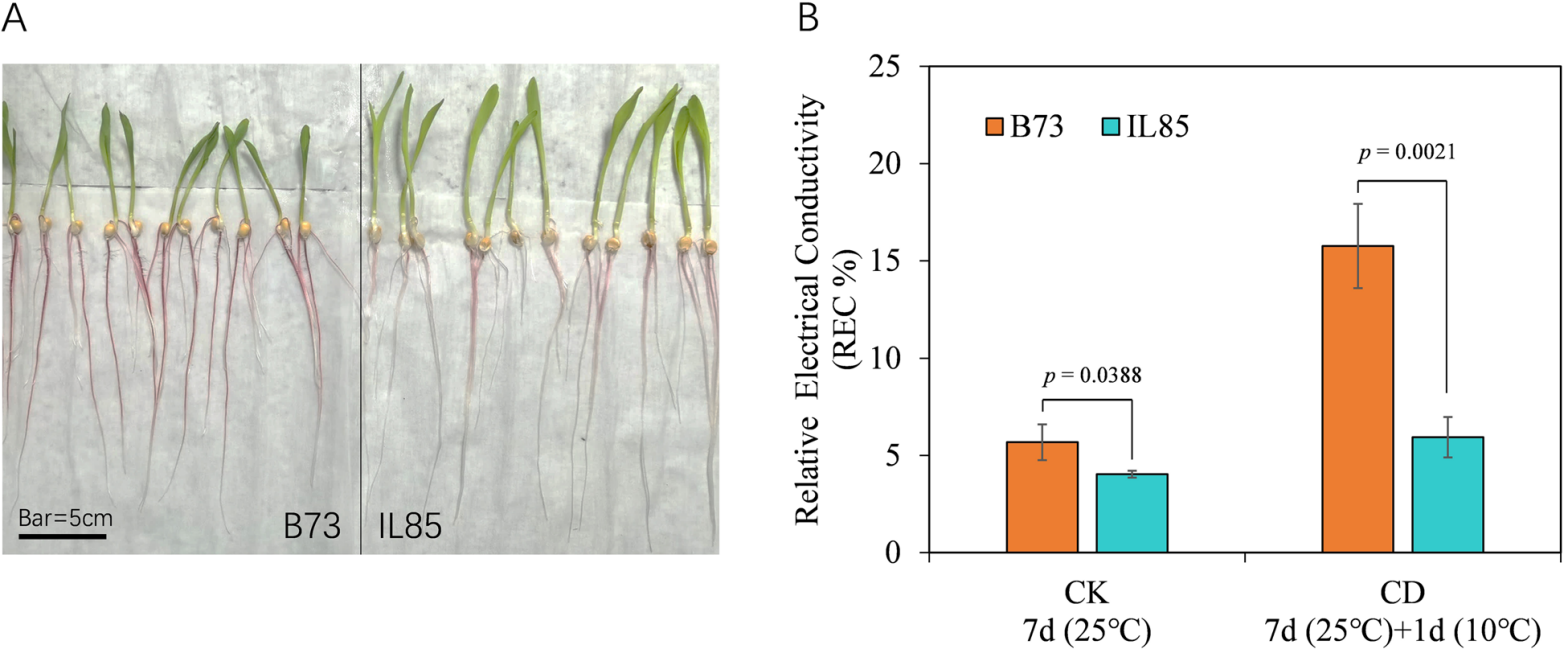
Seedlings and the relative electrical conductivity (REC) of root tips of maize seedlings under cold stress. **A** Seedlings of IL85 and B73 at 7 days after germination, bar = 5 cm; **B** REC of root tips of maize seedlings before and after 24 h of cold stress

Based on germplasm screening, IL85 and B73 were regarded as cold tolerant and sensitive lines, respectively. Electrolyte leakage frequently serves as an indicator of cold stress, as the membrane is the first organelle to sense cold stress. To evaluate the electrolyte leakage caused by cold stress, the root tips of the seedlings that were grown at the control temperature for 7 days, and then transferred to cold conditions for 24 h were used to measure the relative electrolyte conductivity (REC) (Fig. 1A). Compared with the control, the REC of root tips of both IL85 and B73 increased after cold stress, as expected. The increase in REC in B73 was more pronounced (approximately 3 times) than that in IL85 (Fig. 1B). These results revealed that IL85 was more resistant to cold than B73.

### Full-length sequencing and transcript expression profiles of 12 samples

To characterize the transcriptomic difference between IL85 and B73 under cold stress, seedlings grown under control or cold conditions were used for root tips collection, library preparation and full-length transcriptome sequencing. A total of 55 Gb of clean data was produced with 3,890,121 to 6,541,618 reads. After removing rRNA sequences, 3,759,176 to 6,309,821 reads were obtained. These reads had an N50 of 695 – 1,265 bp, a mean length of 661 – 1,089 bp, and a maximum length of 14,970–233,119 bp. The number of full-length reads, with primers at both ends of the read, ranged from 3,297,036 to 5,744,254, with an N50 of 552 – 1,122 bp, a mean length of 460–881 bp, and a maximum length of 6,950–11,677 bp, among which 93.7% to 96.9% were mapped to the reference genome (Table 1). Thanks to the long reads produced by the ONT sequencing platform, we identified 25,309 novel transcripts and 4,316 novel genes (Table S1, Dataset 1 and Dataset 2).

**Table 1:** Summary of the results from full-length sequencing of 12 samples

Sample ID^a^	Clean Data					Full-length reads^c^				
	Total reads number	Clean Number^b^	N50 (bp)	Mean length (bp)	Max length (bp)	Reads number	N50 (bp)	Mean length (bp)	Max length (bp)	Mapped reads (Rate)
BCD1	4,298,610	4,127,858	1,183	1,036	24,701	3,609,340	1,034	824	11,258	3,498,849 (96.9%)
BCD2	4,552,299	4,382,518	1,126	984	58,131	3,869,358	986	775	9,704	3,736,319 (96.6%)
BCD3	4,812,931	4,624,033	980	886	42,651	4,101,426	828	682	6,950	3,938,757 (96.0%)
BCK1	6,541,618	6,309,821	695	661	25,330	5,744,254	552	460	8,480	5,421,952 (94.4%)
BCK2	5,594,616	5,389,905	732	696	26,878	4,840,205	590	496	9,278	4,596,506 (95.0%)
BCK3	5,800,487	5,586,539	821	750	95,008	5,030,650	678	547	8,644	4,779,199 (95.0%)
ICD1	4,654,566	4,471,028	1,002	906	71,935	4,051,667	862	697	11,677	3,861,154 (95.3%)
ICD2	3,890,121	3,759,176	1,265	1089	15,250	3,297,036	1,122	881	10,402	3,173,572 (96.3%)
ICD3	4,797,794	4,620,255	1,034	932	14,970	4,085,571	891	722	10,777	3,903,260 (95.5%)
ICK1	6,126,592	5,906,096	750	718	233,119	5,316,572	605	513	9425	4,983,658 (93.7%)
ICK2	6,119,503	5,872,403	698	680	202,515	5,331,339	550	476	10,613	4,998,266 (93.8%)
ICK3	5,523,159	5,322,332	833	772	85,786	4,792,444	684	567	8969	4,527,555 (94.5%)

^a^Sample ID: inbred line (B and I represent B73 and IL85 respectively) + treatment (CK and CD represent control and cold stress respectively) + replication;

^b^Number of reads after eliminating rRNA;

^c^Reads with primers at both ends

**Fig. 2:**
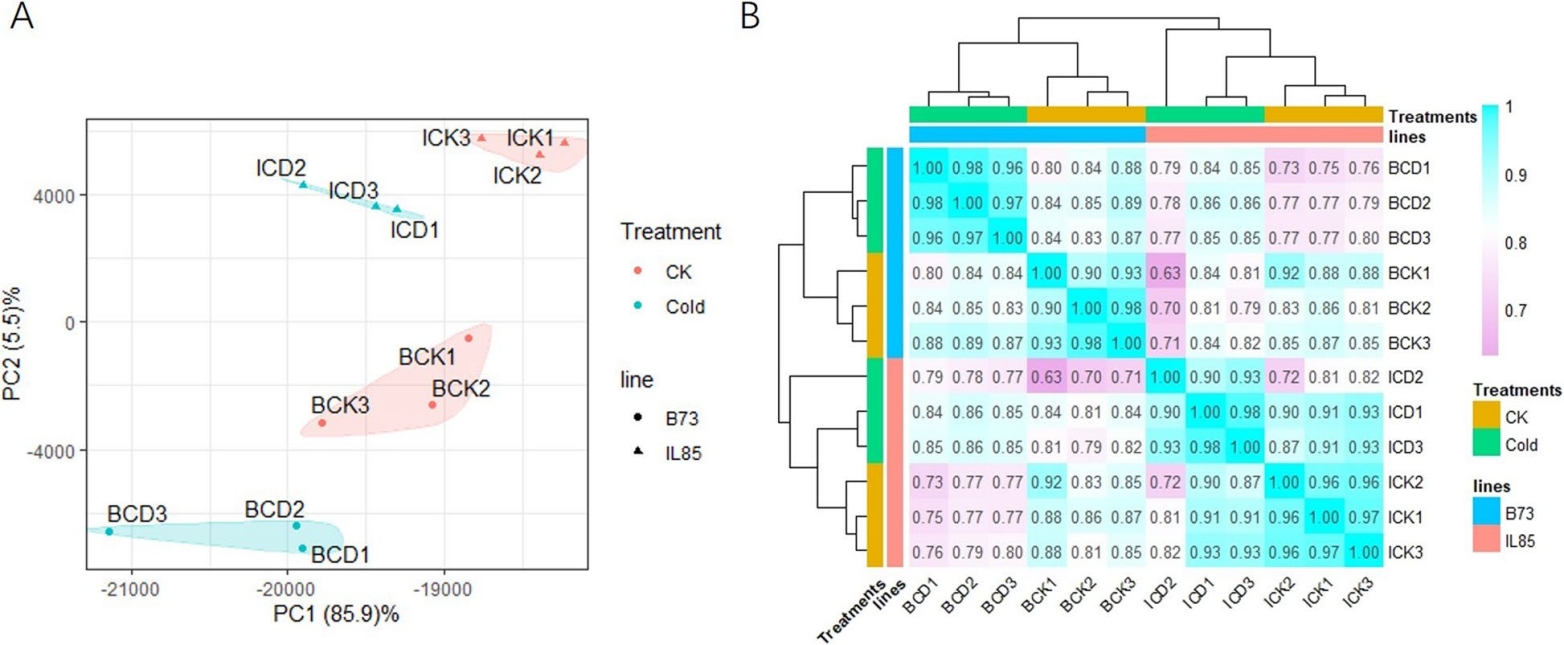
Comparison of transcript expression profiles from 12 samples. **A**, Principal component analysis (PCA) of 12 samples; (**B**), Hierarchical clustering and correlation coefficient based on transcripts expression of each samples. Sample ID named following inbred line (B and I represented B73 and IL85 respectively) + treatment (CK and CD represented control and cold stress respectively) + replication

Principal component analysis (PCA) and hierarchical clustering analysis based on the counts per million (CPM) of all transcripts in 12 samples revealed a major genotypic effect as well as an effect of treatment. Replicate samples clustered together and showed a reasonably high correlation as expected (Fig. 2). Furthermore, both control and cold-treated samples of B73 trended to cluster together, as did those of IL85 (Fig. 2B).

### DET identification and characterization

**Fig. 3:**
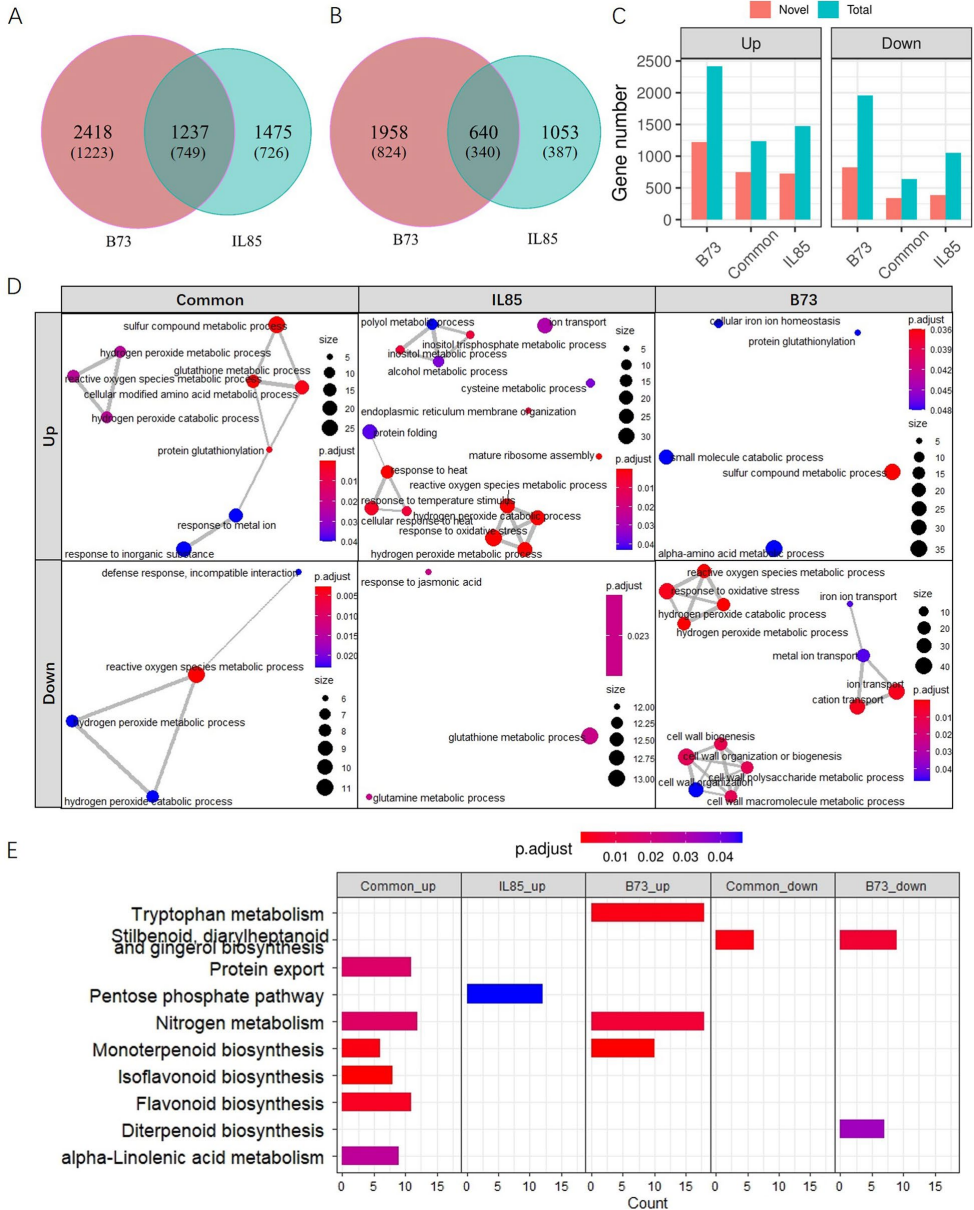
Differentially expressed transcript (DET) identification and functional analysis. Venn diagram for up- (**A**) and down-regulated (**B**) DETs for B73 and IL85, where the number in brackets indicates novel identified transcripts. **C** Statistics of the identified DETs for B73 and IL85; (**D**), GO enrichment analysis for DETs that were specific or common to B73 and IL85 in the biological process (BP) category, where a dot indicates a GO term, the dot size indicates the transcript number belonging to an individual GO term, the dot colour indicates the p-value of the GO term, and the width of line between two dots indicates the common transcript number of two GO terms. **E** Kyoto Encyclopedia of Genes and Genomes (KEGG) pathway enrichment analysis for DETs that were specific or common to B73 and IL85

To assess the transcriptome changes under cold stress, DETs with at least a twofold difference in expression and a p value less than 0.01 were identified for two comparisons, BCK v.s BCD and ICK v.s ICD. A total of 3,655 and 2,598 up- or down-regulated DETs were identified in B73. For IL85, 2,712 and 1,693 up- or down-regulated DETs were identified. Further analysis using a Venn diagram showed that both unique and overlapping DETs were detected (Fig. 3A-C). For up-regulated DETs, a total of 2,418 and 1,475 DETs were unique to B73 and IL85, and 1,237 DETs were commonly differentially expressed in both lines. Regarding down-regulated DETs, a total of 1,958 and 1,053 DETs were unique to B73 and IL85, respectively, and 640 DETs were commonly differentially expressed in both lines. Among these DETs, more than 50% were novel transcripts (Fig. 3A-C). Among these DETs, a total of 982 could be recognized as transcription factors (TFs), 591 or 391 of which were up- or down-regulated in both lines after 24 h cold stress (Table S2).

### Functional analysis of DETs

The common or specific DETs might participate in the basic or genotype-specific cold response process. To analyse the function of these DETs, GO enrichment analyses were performed. GO enrichment analysis of DETs revealed that these transcripts were related to various functions in the biological process (BP) category (Fig. 3D). For up-regulated transcripts under cold stress, DETs that were common to both B73 and IL85 were mainly related to reactive oxygen scavenging, sulfur compound or amino acid metabolic processes. Transcripts that were specifically differentially expressed in IL85 were also related to reactive oxygen scavenging and other GO terms, such as response to heat and inositol. Transcripts specific to B73 were mainly involved in sulfur compound metabolic process, alpha-amino acid metabolic process and so on. For down-regulated transcripts under cold stress, the DETs that were common to both B73 and IL85 were mainly related to reactive oxygen species scavenging. Furthermore, the same reactive oxygen species scavenging related GO terms were identified for transcripts that were specifically down-regulated in B73. Transcripts that were specifically expressed in IL85 were mainly related to glutathione metabolic process, glutamine metabolic process and response to jasmonic acid.

To identify the molecular pathways underlying cold stress, KEGG pathway enrichment analysis was performed on the specific and common DETs mentioned above. These transcripts were enriched in a total of ten pathways (Fig. 3E). The common up-regulated DETs in both IL85 and B73 were related to six pathways. Transcripts related to the pentose phosphate pathway were specifically up-regulated in IL85 under cold stress. B73 specific up-regulated transcripts were enriched in three pathways, such as tryptophan metabolism. For down-regulated transcripts, the diterpenoid biosynthesis pathway was identified and was unique to B73, but no pathway was detected for IL85.

The expression patterns of key transcripts in IL85 under cold stress were investigated. There were 67 transcripts related to three kinds of functions, including 34 for reactive oxygen species scavenging, 21 for response to heat and 12 for inositol (Table S3). The heatmap revealed that the expression of these transcripts was relatively lower in B73 than in IL85 under both conditions. After 24 h of cold stress, the expression of these transcripts rapidly increased in IL85, while they slightly decreased in B73 (Fig. S1).

### Characterization of alternative splicing events

**Fig. 4:**
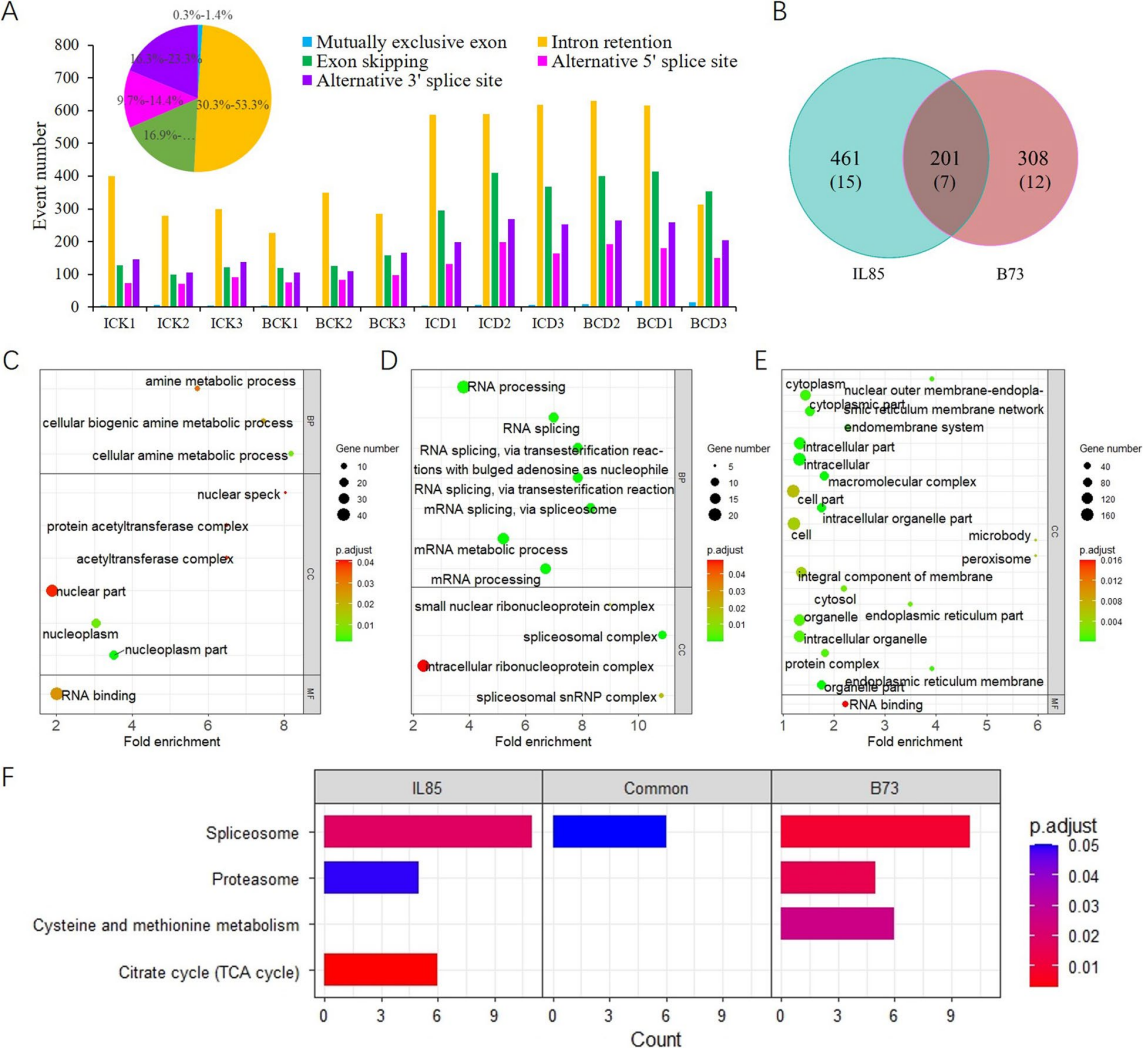
Identification of alternative splicing (AS) events and functional analysis. **A** Statistics of the number of AS events in each sample. The pie diagram reveals the proportion of five AS events. **B** A Venn diagram of the differential AS between B73 and IL85. GO enrichment analysis for DAS related genes specific to IL85 (**C**), common to both lines (**D**) and specific to B73 (**E**), respectively. The dot size indicates the number of genes, the dot color indicates the *p*-value of GO term. **F** DAS related genes specific to IL85, common to both lines and specific to B73. BP, biological process; CC, cellular component; MF, molecular function

Alternative splicing (AS) is a key step in plant abiotic stress acclimation. Identifying and characterizing AS events enhances our understanding of the biological role of transcript isoform diversity. In this study, five categories of AS events were identified in 12 samples, the most abundant event was intron retention (30.3%-53.3%), and the least abundant event was mutually exclusive exons (0.3%-1.4%). The number of AS events was higher in samples under cold stress than under the control temperature, especially intron retention events (Fig. 4A). The AStalavista tool was also used to detect differential AS (DAS) events between samples with or without cold stress in each inbred line. A total of 662 and 509 genes with DAS (DAS genes) were identified for IL85 and B73, respectively. There were 201 genes that showed DAS in both lines, and a total of 461 and 308 DAS genes were specific to IL85 and B73, respectively (Fig. 4B). Interestingly, the common DAS genes were significantly enriched in seven GO terms in the BP category, and all these terms were related to RNA processing, as expected (Fig. 4C). The DAS genes uniquely identified in IL85 were mainly enriched in three amine metabolic process related GO terms (Fig. 4D), and the specific DAS genes for B73 were not significantly enriched in any GO terms in the BP category (Fig. 4E). KEGG enrichment analysis of these DAS genes was used to explore their molecular pathways. A total of four pathways were detected, and all three DAS gene sets were enriched in the spliceosome. The TCA cycle was found to be a unique pathway in IL85, while cysteine and methionine metabolism was unique to B73 (Fig. 4F). The results presented here suggested that the AS of genes played an important role in the maize cold stress response and that there were obvious genotypic differences.

Eight DAS genes that were unique to IL85 were related to amine metabolic process according to GO enrichment analysis. These genes contained 126 transcripts including 40 novel transcripts (Table S4). The expression of each transcript is shown in Figure S2A. More than one-third of the transcripts were predominantly expressed in IL85 root tips under cold stress, and seven out of eight genes had at least one transcript that was predominantly expressed in ICD samples. To further investigate the AS event, sashimiplots were generated for Zm00001d019698 and Zm00001d053404 using igv tools. Zm00001d019698 encodes a cyclase-like protein 4 with 7 exons, and many reads mapped to the beginning of the third intron in B73 (Fig. S2B), which suggested that different intron retention events occurred between the two genotypes. The gene Zm00001d053404 has 13 transcripts, and encodes a spermidine synthase. Additionally, because of intron retention events, six novel transcripts were identified in B73 under cold stress (Fig. S2C). These results suggested that the genotype- and stress-specific splicing isoforms might play an important role in maize cold tolerance.

### Characterization of lncRNAs

**Fig. 5:**
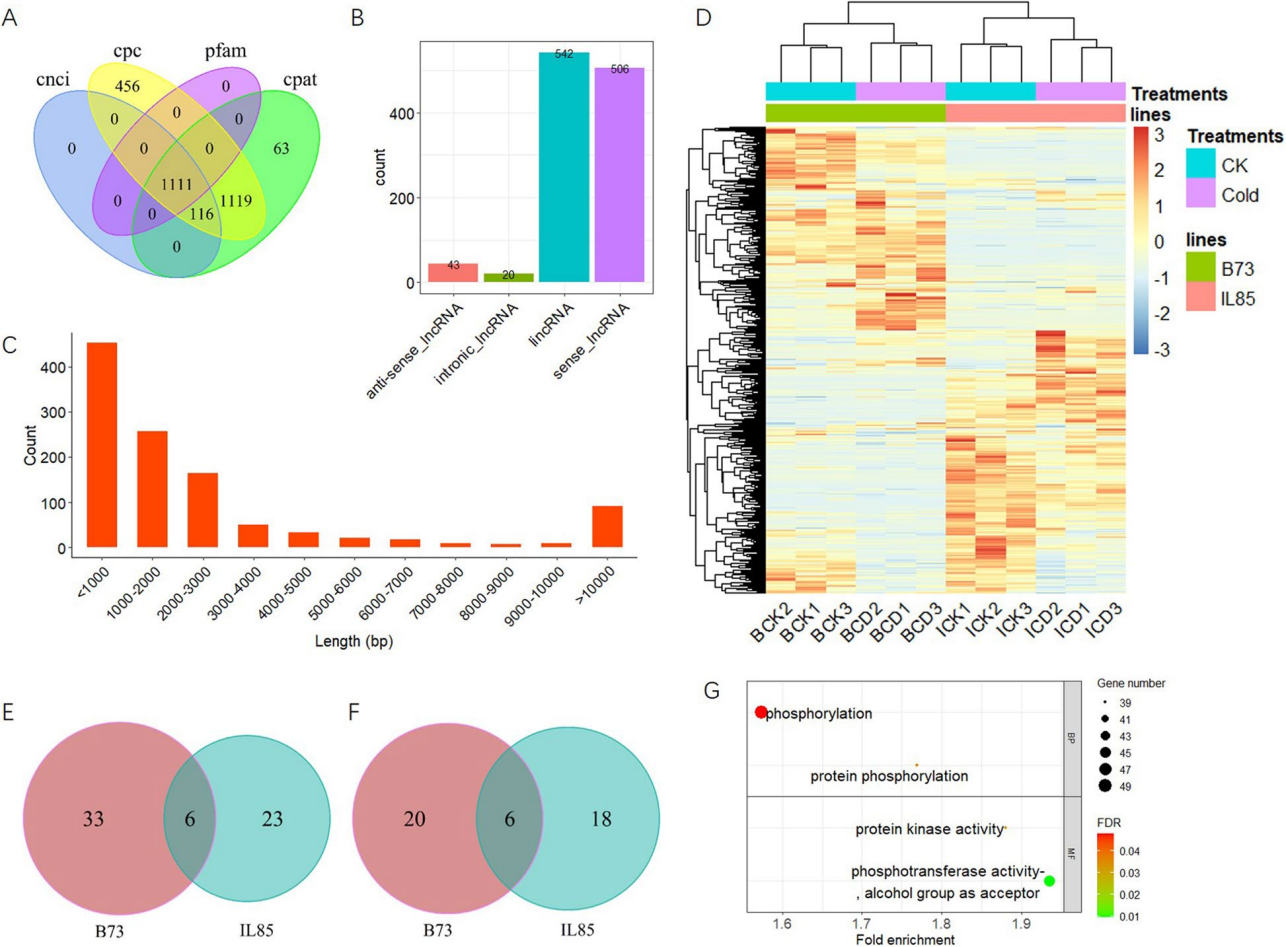
Identification and characterization of long noncodin RNAs (lncRNAs). **A** A Venn diagram of the lncRNAs identified by four different tools. **B** Statistics of location of the lncRNAs. **C** The length distribution of lncRNAs. **D** Hierarchical clustering and heatmap of lncRNAs based on expression level. Venn diagrams of differential lncRNAs that were up- (**E**) and down-regulated (**F**) in B73 and IL85. **G** GO enrichment analysis for target genes of lncRNAs that specifically up-regulaed in B73 or IL85 under cold stress, where the dot size indicates the number of genes, and the dot colour indicates the p-value of the GO term

Long noncodling RNAs (lncRNAs) were identified by performing CNCI, CPC, CPAT and Pfam analysis, and a total of 1,111 lncRNAs were detected by all four methods (Fig. 5A and Dataset 3). These lncRNAs grouped into four categories with 43 anti-sense lncRNAs, 20 intronic lncRNAs, 542 lincRNAs, and 506 sense lncRNAs (Fig. 5B). The length of most of the lncRNAs was less than 3000 bp (Fig. 5C). The expression profiles of lncRNAs are shown in Fig. 5D. All lncRNAs can be divided into two major groups, those mainly expressed in B73 or IL85, and there were differences in lncRNA expression patterns between treatments within each genotype (Fig. 5D). The expression differences of each lncRNA were calculated by the DEseq2 package. With a cut-off of an absolute value of log2FC > 1 and *p*-value <  = 0.01, a total of 106 lncRNAs showed a significant difference between treatments within each genotype, with 62 up-regulated (Fig. 5E), and 44 down-regulated lncRNAs (Fig. 5F). For up-regulated lncRNAs, 33 and 23 were specifically expressed in IL85 and B73, and 6 lncRNAs were commonly expressed in both IL85 and B73. Among the down-regulated lncRNAs, 20 and 18 were specific to IL85 and B73, respectively, and 6 lncRNAs were common to IL85 and B73.

To explore the potential function of lncRNAs, their target genes were predicted according to their position and complementary sequences. A total of the 9069 genes were predicted to be targets of these 1,111 lncRNAs, including 3,580 cis- targeted and 5,489 trans-targeted genes (Dataset 4). For the 62 up-regulated lncRNAs in IL85 or B73, there were 495 corresponding target genes, which were mainly involved in the phosphorylation process according to GO enrichment analysis (Fig. 5G). There were 572 target genes for the 44 down-regulated lncRNAs, and no significant GO terms or KEGG pathways were identified by enrichment analysis, indicating that down-regulated lncRNAs might not participate in particular biological processes or be involved in more extensive processes.

### Validation of the expression of selected genes by Quantitative Real-Time PCR (qRT-PCR)

**Fig. 6:**
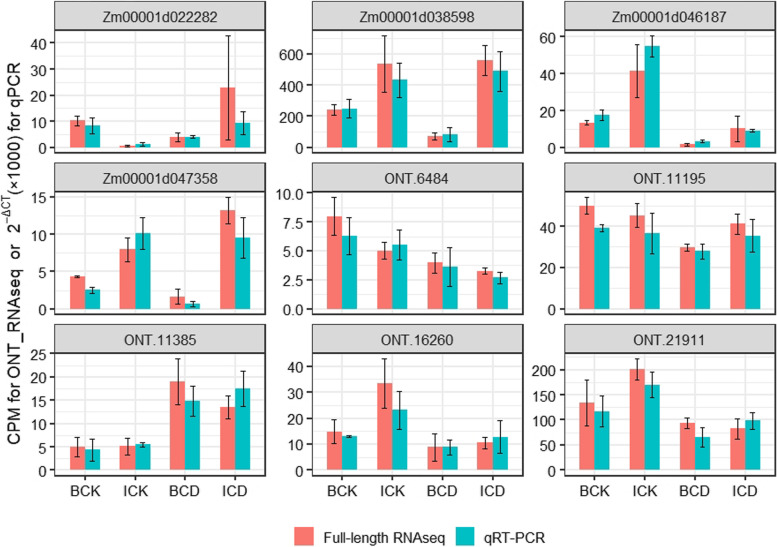
Verification and comparison of full-length RNA-seq and qRT-PCR. *ZmTub* was used as an internal control. Values and bars represent the mean and standard error of three biological replicates, respectively. CPM indicates counts per million; ONT indicates Oxford Nanopore technologies

The expression of ten genes, including five novel genes, four known genes and an endogenous control gene (*ZmTub*), was quantified with qRT-PCR to validate the results of ONT RNA sequencing (Fig. 6). As expected, there was a similar tendency and a high correlation between the results of qRT-PCR and the ONT RNA sequencing global profiling (Fig. S3), demonstrating the robustness of the RNA sequencing results.

## Discussion

Abiotic stress leads to substantial changes in the transcriptome in plants. To precisely characterize gene expression patterns and molecular features under cold stress, several studies have assessed transcriptome changes at the germination and seedling stages using the Illumina sequencing platform, and hundreds of genes in response to cold stress have been identified [12–20]. In maize, approximately 50% of genes contain more than 4 exons, with an average of 3.3 transcripts per gene [28]. Given that mRNA transcripts are straightforward template for translation, identifying the changes in transcript abundance under cold stress is the main task. In this study, we performed full-length RNA sequencing on the root tips of seedlings with or without cold stress in maize. A total of 152,263 transcripts were detected, including 20,993 novel transcripts (Table S1). We identified 3,655 up-regulated DETs in IL85 under cold stress through comparative transcriptome analysis (Fig. 3A), these DETs were mainly involved in the ROS scavenging process (Fig. 3D). Alternative splicing (AS) events are markedly induced in response to environmental stress, allowing rapid adjustment of the abundance and function of key stress-response components [29]. Long read lengths contributed to the identification of AS events. A total of 970 DAS events were detected in response to cold stress, and the overlapping set of DAS involved genes was mainly related to RNA splicing or spliceosome, as expected (Fig. 4D and F). The DAS genes unique to IL85 were related to the amine metabolic process (Fig. 4C). LncRNAs have been shown to be involved in the response to cold stress [30–32]. We also identified 62 and 44 lncRNAs that were up- and down-regulated in both lines under cold stress, and the target genes of up-regulated lncRNAs were mainly involved in protein phosphorylation (Fig. 5G), which is consistent with the findings of others [32]. These results suggested that three analysis methods, the identification of DETs, AS events and differential lncRNAs, reflected different aspects of transcriptome response to cold stress, and no single analysis can capture the complete view.

### ROS scavenging enzymes played an important role in maize cold acclimation

**Fig. 7:**
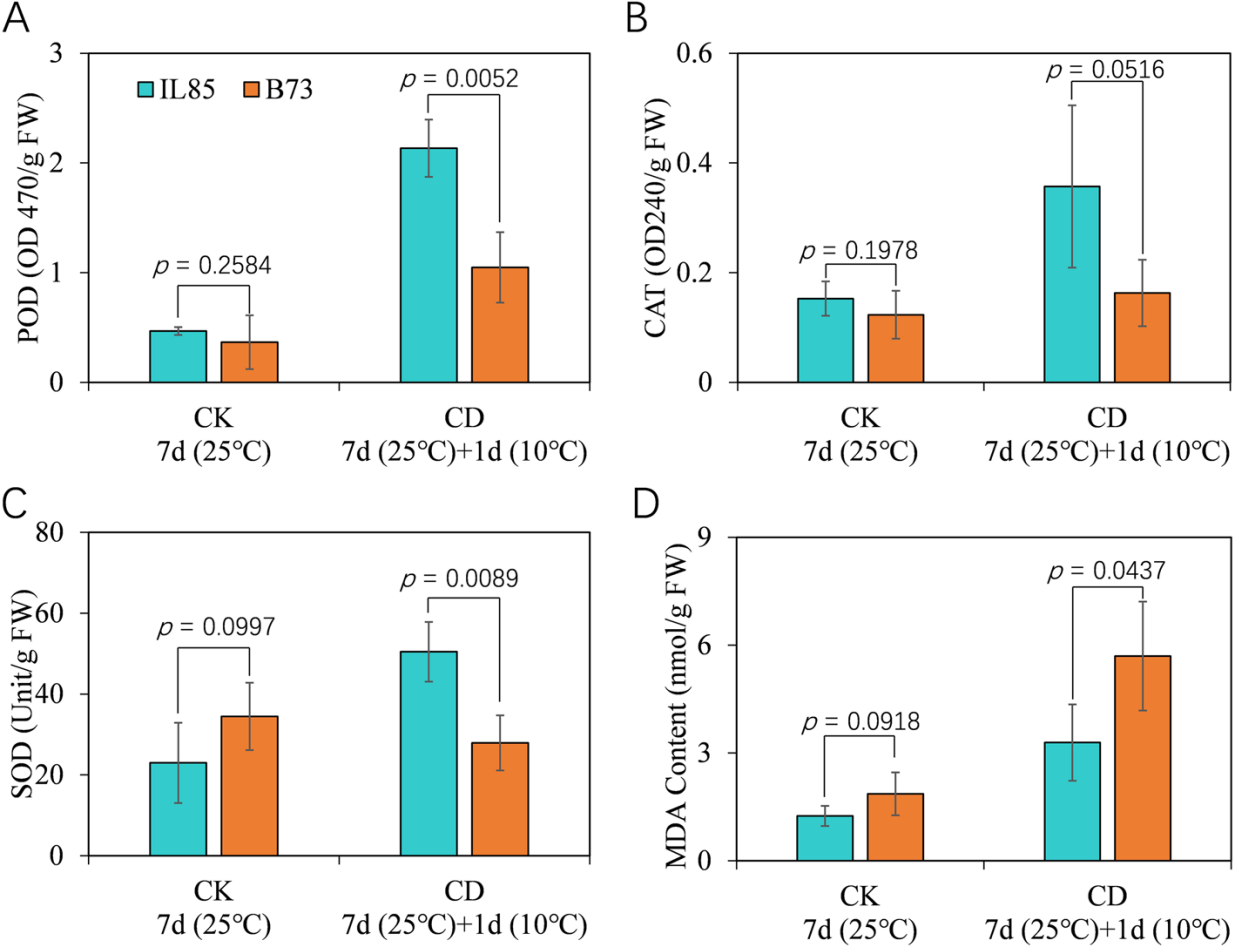
The activities of three antioxidant enzymes and the content of MDA in the root tips with or without 24 h of cold stress. The activities of POD (**A**), CAT (**B**) and SOD (**C**), respesitively. **D** the content of MDA

ROS can be generated as a consequence of electron leakage during photosynthesis and respiration in cells. Superoxide radicals, hydrogen peroxide, hydroxyl radicals, and singlet oxygen are the main ROS formed in response to the reduction of oxygen molecules in planta [33]. The production and removal of ROS must be strictly controlled, because ROS cause lipid peroxidation, which leads to the loss of plasma membrane integrity and electrolyte leakage [34–38]. However, the equilibrium between the production and scavenging of ROS may be perturbed by numerous adverse abiotic stress factors, such as cold stress [39]. To moderate intracellular ROS homeostasis, plant have evolved an enzymatic antioxidative mechanism, including superoxide dismutase (SOD), catalase (CAT), and peroxidase (POD). In this study, a total of 34 transcripts that related to ROS scavenging enzymes were predominantly expressed in IL85 under cold stress, 25 of which encode peroxidase, while transcripts with similar function were significantly down-regulated in B73. This is perhaps the principal reason that the REC of IL85 is much lower than that of B73 under cold stress (Fig. 1B). To illustrate this, we have measured the activities POD, CAT and SOD, the results show that the activities of all the three antioxidant enzymes in IL85 were higher than that in B73, although the activity of POD was also increased after 24 h of cold stress in B73 (Fig. 7A to 7C). As a result, changes in antioxidant enzymes would alter the cellular redox homeostasis. Malondialdehyde (MDA) is the best investigated product of lipid peroxidation induced by oxidative stress [40], it could be served as an indirect indicator of ROS level. Then the content of MDA was measured, and it was lower in IL85 root tips than that in B73 (Fig. 7D) as expected, which may explain part of the different REC between IL85 and B73 under cold stress (Fig. 1B). It has been revealed that the activities of SOD, POD and CAT increase in *X. sorbifolia* under cold stress [41]. The increase in antioxidant enzyme activity was the main reason for the enhanced cold tolerance in transgenic rice (*GmFAD3A*) [42], as several POD genes were dramatically induced after cold treatment. The expression of these genes increased in the overexpression plants (*PtrbHLH*) but decreased in the RNAi plants of trifoliate orange [43]. As a result, the accumulation of ROS scavenging enzymes was appreciably alleviated in the transgenic lines of trifoliate orange under cold stress [44].

### Amine metabolism and maize cold tolerance

Polyamines (PAs) are the main forms of amine-containing compounds in plants, and are elevated under cold stress. They have dual effects, such as being protectors and perpetrators of stress damage to the cells. It has been demonstrated that PAs play a role in providing cold tolerance [45, 46]. In this work, we found that amine metabolism related genes showed specific DAS events (Fig. 4C), and some transcripts were predominantly expressed in IL85 under cold stress (Fig. S2A). Compared with the other treatments, putrescine (Put) and spermidine (Spd) levels were much greater under cold stress [47]. Putrescine (Put) has been shown to accumulate in the leaves of tomato but to be higher in chilling-tolerant cultivar than in chilling-sensitive cultivars [48]. The rate of polyamine (spermine and spermidine) synthesis and the levels of transcripts encoding arginine decarboxylase, SAMDC and spermine/spermidine synthase also increased after cold exposure [49–51]. A naturally cold-tolerant variety of rice showed higher expression of SAMDC genes [50]. Loss of function of *ADC1* and *ADC2*, two key genes for polyamine metabolism, reduced cold tolerance in Arabidopsis [52]. Increasing the content of PAs contributed to alleviating chilling stress in cucumber and tomato seedlings when melatonin was applied [48, 53]. These results suggested that understanding the role of the PA metabolism at the molecular level will be pivotal for improving maize performance under cold stress conditions.

### Polyamine oxidases involved in ROS and PA metabolism

The accumulation of ROS was frequently accompanied by an increase in polyamines during cold acclimation [46]. ROS, such as H_2_O_2_, can be produced by polyamine catabolism processes. In plants, polyamine oxidases (PAOs) catalyse the terminal catabolism (TC) of Spm and Spd and produce N-(3-aminopropyl)-4-aminobutanal from Spm and 4-aminobutanal from Spd, along with 1,3-diaminopropane (DAP) and H_2_O_2_ [54–56], PAO is the most important enzyme in polyamine homeostasis and plays an essential role in growth and developmental processes and responses to abiotic stresses [57]. In this study, we found that at least one of the polyamine oxidase encoding transcripts was more highly expressed in IL85 under cold stress (Fig. 4C). In rice, at least three PAOs (*OsPAO4/6/7*) were induced by cold treatment, while some PAOs were invariable or even decreased under cold stress [58, 59]. In turn, *OsPAO2* and *OsPAO6* were strongly induced upon oxidative stress (H_2_O_2_) [58].

## Conclusions

In summary, we characterized the transcript changes related to early cold stress and the initiation of cold acclimation using full-length RNA sequencing. By comparing the full-length transcriptome between seedlings subjected to cold stress across two maize inbred lines (IL85 and B73), we suggested that transcripts involved in ROS scavenging enzymes and amine metabolism played an important role in cold acclimation. These results provide new insights into the molecular mechanism of cold acclimation at the seedling stage in maize, and facilitate to development of cultivars with improved cold stress tolerance.

## Methods

### Plant materials

According to our previous experiment, the maize inbred line IL85 was selected as a cold tolerant line, and B73 was used as a cold susceptible line. The seeds of both lines were harvested from the experimental base of the Institute of Nanfan & Seed Industry after self-pollination. Then, the seeds, sterilized with 1% sodium hypochlorite for 5 min, were placed on moist germination paper and another sheet of moist paper was used as a cover. The two pieces of germination paper were rolled together and placed vertically in a sealed plastic bag [60]. After germinating at 25 °C/22 °C with an 8 h light/16 h dark cycle for 7 days, the root tips (bottom ~ 2 cm) of the primary roots were sampled. Then, the remaining paper rolls were transferred to an incubator with a constant temperature of 10 °C with an 8 h light/16 h dark cycle for 24 h, and the root tips were harvested. Each sample was divided into two equal parts. One part was immediately stored in liquid nitrogen for total RNA extraction, and the remaining part was used for electrical conductivity measurement.

### Determination of electrolyte leakage

A total of 15 freshly harvested root tips were incubated at room temperature in tubes with 15 mL of distilled water for 20 min. Then, the electrical conductivity (EC1) of the solution was measured using a conductivity meter (Mettler Toledo). The tubes were heated at 100 °C for 15 min. After cooling to room temperature, the electrical conductivity (EC2) was determined. The final relative electrical conductivity (REC) was estimated according to the equation [61]:

$$\mathrm{REC }= (\mathrm{EC}1/\mathrm{EC}2) \times 100$$

### RNA extraction, library preparation, and Oxford Nanopore Full-Length Sequencing

Root tips stored in liquid nitrogen were pooled and ground in liquid nitrogen for total RNA extraction using the RNAprep Pure Plant Kit (Tiangen, China). Then, 1 ug of total RNA was prepared for cDNA libraries using a cDNA-PCR Sequencing Kit (SQK-PCS109) according to the manufacturer’s protocol (Oxford Nanopore Technologies, US). The final cDNA libraries were added to FLO-MIN109 flowcells and run on the PromethION platform at Biomarker Technology Company.

### Read quality control, alignment, transcript expression quantification and differentially expressed transcript (DET) identification

For each sample, raw reads were first filtered with the cutoff of minimum average read quality score = 7 and minimum read length = 500 bp, and the ribosomal RNAs were removed after mapping to the rRNA database. Second, full-length non-chemiric (FLNC) transcripts were identified by searching for primers at both ends of the reads. Clusters of FLNC transcripts were obtained after mapping to the B73 reference genome (GCA_000005005.6_B73_RefGen_v4) with mimimap2 [62], and consensus isoforms were obtained after polishing within each cluster by the pinfish tool. The mapped consensus reads were further collapsed by the cDNA_Cupcake package with min-coverage = 85% and min-identity = 90%. A 5’ difference was not considered when collapsing redundant transcripts. Per-transcript expression levels were determined based on reads with a match quality above 5. Expression levels were estimated by read counts per gene/transcript per 10,000 reads mapped (CPM) in each sample, allowing direct comparison across samples.

The DESeq2 package (1.6.3) was employed to identify differentially expressed transcripts (DETs) between two samples [63]. The resulting P-values were adjusted using Benjamini and Hochberg’s approach for controlling the false discovery rate. Genes/transcripts with an FDR < 0.01 and fold_change ≥ 2 were considered significantly differentially expressed.

### Transcription factors identification

All maize transcription factors (TFs) were identified in Plant Transcription Factor Database (Plant TFDB, http://planttfdb.gao-lab.org/index.php?sp=Zma) [64] and GRASSIUS (https://grassius.org/tfomecollection.php) [65]. A gene was regarded as TF if it was in any of databases, then the corresponding transcripts of TF encoding genes were extracted.

### Alternative splicing event analysis

Transcripts were validated against known reference transcript annotations with gffcompare [66]. AS events, including intron retention (IR), exon skipping (ES), alternative 5’ splice site (A5SS), alternative 3’ splice site (A3SS) and mutually exclusive exon (MEE), and the differential AS events between two samples were detected by the AStalavista tool [67].

### LncRNA analysis

Four computational approaches, CPC/CNCI/CPAT/Pfam, were combined to sort long noncoding RNA candidates from putative protein-coding RNAs in the transcripts [68–71]. Putative protein-coding RNAs were filtered out using a minimum length and exon number threshold. Transcripts with lengths greater than 200 nt and with more than two exons were selected as lncRNA candidates and further screened using CPC/CNCI/CPAT/Pfam, which has the power to distinguish protein-coding genes from the noncoding genes. The differential lncRNAs were identified using DESeq2 (1.6.3) as in the identification of DETs.

### Functional annotation and enrichment analysis

Gene/transcript functions were annotated based on the Gene Ontology (GO) [72, 73] and Kyoto Encyclopedia of Genes and Genomes (KEGG) databases [74–76]. We performed GO and KEGG enrichment analyses of DEGs/DETs based on hypergeometric tests using the phyper function in R software [77], and the resulting p-values were adjusted using Benjamini and Hochberg’s approach for controlling the false discovery rate. The results of enrichment analysis were visualized by the enrichplot and ggplot2 packages.

### Quantitative Real-Time PCR (qRT-PCR) analysis

The expression of ten randomly selected genes, including five novel genes, four known genes and an endogenous control gene, was validated by qRT-PCR. To normalize the relative expression levels for each gene, maize *ZmTub* served as an endogenous control. The primers used for qRT-PCR were designed by the web-based software Primer3 (https://bioinfo.ut.ee/primer3/) and their details are shown in Table S5. cDNA was synthesized from the total RNA prepared for transcriptome sequencing. qRT-PCR MasterMix (Applied Biological Materials Inc.) was used for expression level analysis according to the manufacturer’s protocol. Three biological replicates were conducted and each biological replicate was technically repeated three times. The 2^−ΔCT^ method was used to calculate the relative abundance of genes.

### The activities of antioxidant enzymes and the content of MDA measurement

The root tips were sampled as described above, the activities of antioxidant enzymes (CAT, POD, SOD) were measured according to Elavarthi and Zeng with minor modification [78, 79]. Briefly, approximately 0.1 g root tips tissues were homogenized with 5 μl of Tris–HCl buffer (pH 7.0) containing 20% glycerinum, 1 mM EDTA,1 mmol/L ASA, 1 mmol/L DTT, 1 mmol/L GSH and 5 mmol/L MgCl_2_ on ice. Then the homogenate was centrifuged at 10000 g for 20 min at 4 °C. The supernatant was collected and used for CAT, POD and SOD activity analysis. CAT activity was defined as the absorbance changes (OD240) per gram of fresh weight per minute, POD activity was expressed the absorbance change (OD470) per gram of fresh weight per minute, and the SOD activity was represented as unit that the amont of SOD causing 50% inhibition of NBT reduction.

The content of MDA was measured using a Solarbio kit (BC0025), the main principle behind this kit was that MDA and TBA could be synthetized trimethine under acidic and high temperature conditions, and the trimethine has a maximum absorbance at 532 nm.

## Supplementary Information

**Additional file 1:** **Figure S1.** Heatmap of transcripts corresponding to three GO function (ROS scavenging, response to heat and inositol)

**Additional file 2:** **Figure S2.** Differential AS events corresponding to amine metabolism

**Additional file 3:** **Figure S3**. Scatter plot revealed the correlation of gene expression characterized by full-length RNAseq and qRT-PCR

**Additional file 4:** **Table S1.** Statistics of genes and transcripts

**Additional file 5:** **Table S2**. Transcription factor encoding DETs that related to cold stress

**Additional file 6:** **Table S3.** ROS scavening, responst to heat and inositol related transcripts

**Additional file 7:** **Table S4.** Important DETs related to cold stress

**Additional file 8:** **Table S5.** Primer used for qRT-PCR

**Additional file 9:** **DataSet1.** Transcript count matrix and annotation

**Additional file 10:** **DataSet2.** Transcript

**Additional file 11:** **DataSet3.** LncRNA

**Additional file 12:** **DataSet4.** LncRNAs and target gene

## Data Availability

The datasets supporting the conclusions of this article is(are) available at NCBI under SRA accession number PRJNA822071.

## Abbreviations

Bp: Basepairs
H: Hour
Min: Minute
ONT: Oxford Nanopore technologies
DET: Differentially expressed transcript
DAS: Differentially alternative splicing
lncRNA: Long noncoding RNA
ROS: Reactive oxygen species
REC: Relative electrolyte conductivity
FLNC: Full-length non-chimeric
PCA: Principal component analysis
CPM: Counts per million
BP: Biological process
CC: Cellular component
MF: Molecular function.
GO: Gene ontology
KEGG: Kyoto encyclopedia of genes and genomes
qRT-PCR: Quantitative real-time PCR
SOD: Superoxide dismutase
CAT: Catalase
POD: Peroxidase
PA: Polyamine
PAO: Polyamine oxidases
IR: Intron retention
ES: Exon skipping
A5SS: Alternative 5’ splice site
A3SS: Alternative 3’ splice site
MEE: Mutually exclusive exon
Put: Putrescine
Spd: Spermidine
MDA: Malondialdehyde
TF: Transcription factor
EDTA: Ethylene diamine tetraacetic acid
ASA: Ascorbic acid
DTT: Dithiothreitol
GSH: Glutathione
NBT: Nitro-blue tetrazolium
TBA: Thiobarbituric acid
