# Supplementary material for: Full-length transcriptome analysis of maize root tips reveals the molecular mechanism of cold stress during the seedling stage
Source: BMC Plant Biol. 2022 Aug 13;22:398. doi: 10.1186/s12870-022-03787-3 (PMC9375949; doi:10.1186/s12870-022-03787-3)
Supplement: Supplementary file 1 — Additional file 1: Figure S1. Heatmap of transcripts corresponding to three GO function (ROS scavenging, response to heat and inositol) [file 12870_2022_3787_MOESM1_ESM.docx]

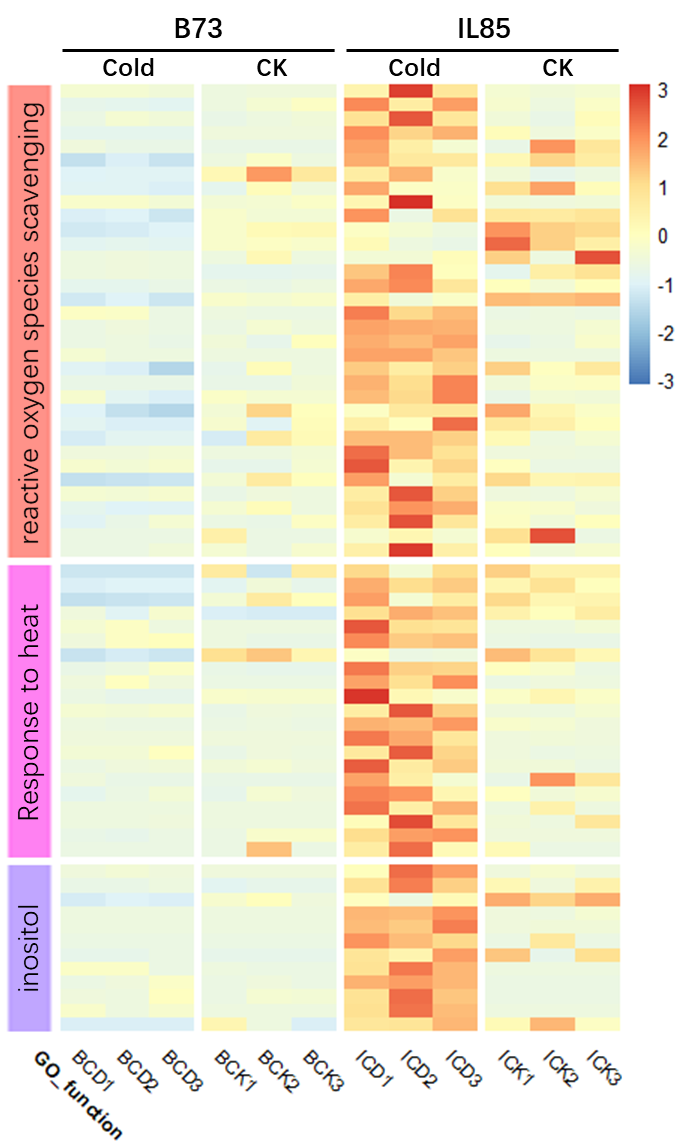


Figure S1. Heatmap of transcripts corresponding to three GO function (ROS scavenging, response to heat and inositol)
