# Supplementary material for: Full-length transcriptome analysis of maize root tips reveals the molecular mechanism of cold stress during the seedling stage
Source: BMC Plant Biol. 2022 Aug 13;22:398. doi: 10.1186/s12870-022-03787-3 (PMC9375949; doi:10.1186/s12870-022-03787-3)
Supplement: Supplementary file 2 — Additional file 2: Figure S2. Differential AS events corresponding to amine metabolism [file 12870_2022_3787_MOESM2_ESM.docx]

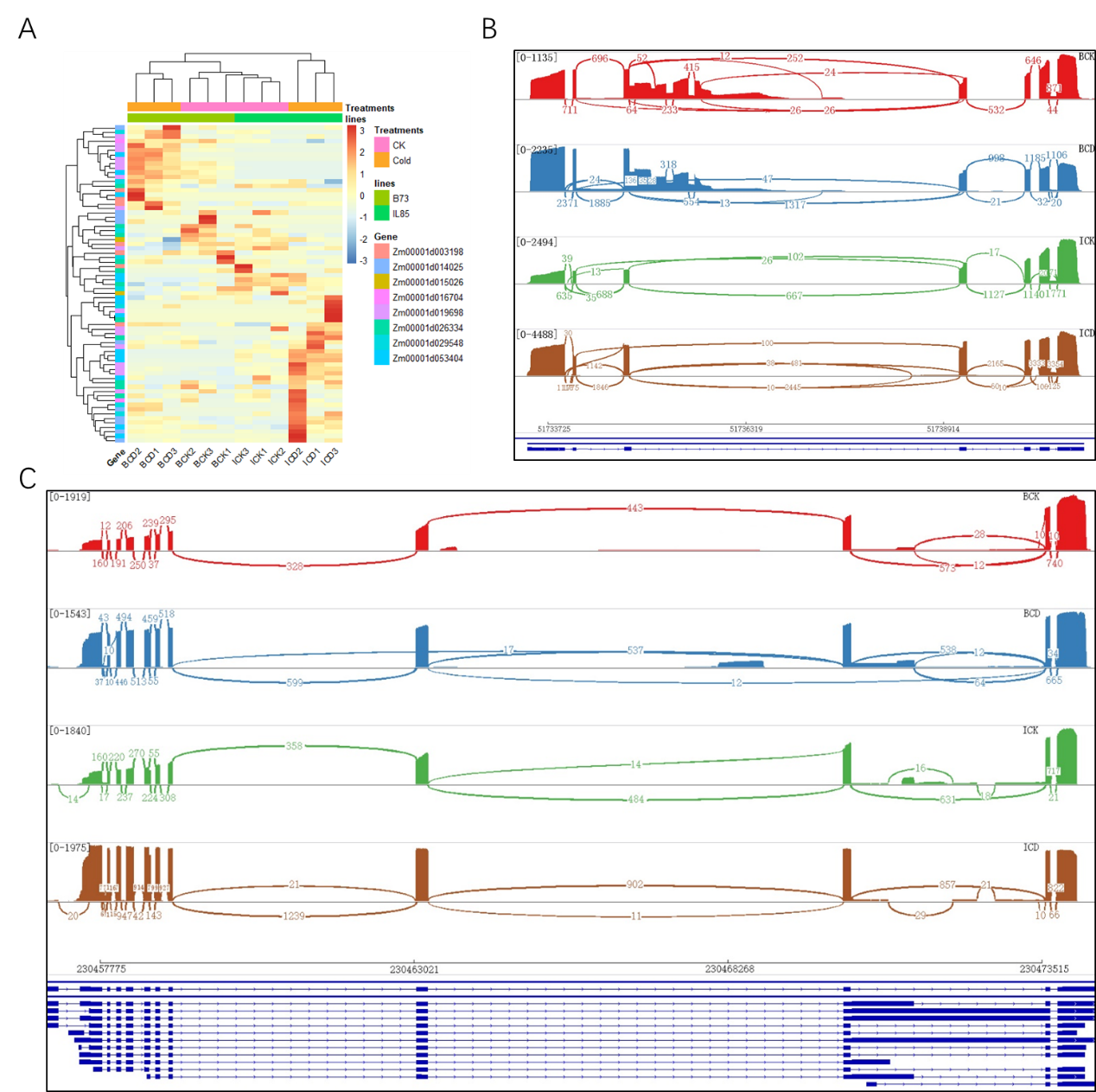


**Figure S2**. Differential AS events corresponding to amine metabolism.

**(A)**, Heatmap of transcripts of eight genes that related to differential AS events corresponding to amine metabolism; **(B)**, Sashimi diagram for Zm00001d019698; C, Sashimi diagram for Zm00001d053404.
