# Supplementary material for: Full-length transcriptome analysis of maize root tips reveals the molecular mechanism of cold stress during the seedling stage
Source: BMC Plant Biol. 2022 Aug 13;22:398. doi: 10.1186/s12870-022-03787-3 (PMC9375949; doi:10.1186/s12870-022-03787-3)
Supplement: Supplementary file 3 — Additional file 3: Figure S3. Scatter plot revealed the correlation of gene expression characterized by full-length RNAseq and qRT-PCR [file 12870_2022_3787_MOESM3_ESM.docx]

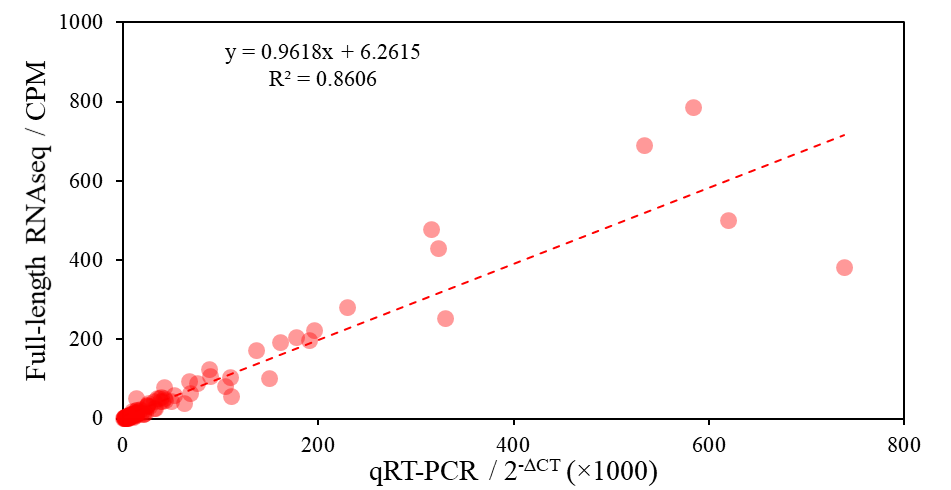


**Figure S3**. Scatter plot revealed the correlation of gene expression characterized by full-length RNAseq and qRT-PCR.
